# Mesenchymal stem cells in the treatment of coronavirus-induced pneumonia (COVID-19)

**DOI:** 10.31744/einstein_journal/2020CE5802

**Published:** 2020-08-25

**Authors:** Angela Mazzeo, Enrico Jardim Clemente Santos

**Affiliations:** 1 Instituto Israelita de Ensino e Pesquisa Albert Einstein Hospital Israelita Albert Einstein São Paulo SP Brazil Instituto Israelita de Ensino e Pesquisa Albert Einstein, Hospital Israelita Albert Einstein, São Paulo, SP, Brazil.; 2 Celltrovet-Células Tronco Veterinárias São Paulo SP Brazil Celltrovet-Células Tronco Veterinárias, São Paulo, SP, Brazil.

Dear Editor,

Stem cell therapy has been investigated by a number of basic, pre-clinical and clinical studies, and these studies have proved that stem cells are safe and effective in the treatment of many medical conditions and diseases.^(1-4)^ A study including 10 patients with confirmed diagnosis for coronavirus 2019 (COVID-19), and pneumonia due to infection by severe acute respiratory syndrome coronavirus 2 (SARS-CoV-2), reported significant improvement in symptoms of all patients treated with mesenchymal stem cells (MSCs) with no adverse effects. Of these patients, 7 were treated with intravenous concentration of 1x10^6^ cells per kilogram of body weight and 3 were treated with placebo. Patients were followed-up for 14 days. Results observed included a significant reduction of chest infiltration, reduction of proinflammatory cytocines including tumor necrosis factor alpha (TNF-α), and an increase of peripheral lymphocytes rate with phenotype recovering of CD4^+^ T cell count and dendritic. In addition, an increase was observed of anti-inflammatory gene expression and trophic factors.^(5)^ Although further studies including more patients are warranted, data published in the literature so far suggest that MSCs is safe and effective for treatment in patients with SARS-CoV-2 pneumonia.^(5)^

Chinese Clinical Trial Registry (ChiCTR2000029990).
