# Supplementary material for: Mesenchymal stem cells in the treatment of coronavirus-induced pneumonia (COVID-19)
Source: Einstein (Sao Paulo). 2020 Aug 25;18:eCE5802. doi: 10.31744/einstein_journal/2020CE5802 (PMC9586424; doi:10.31744/einstein_journal/2020CE5802)
Supplement: Translation

# Translation: Células-tronco mesenquimais no tratamento da pneumonia induzida pelo coronavírus (COVID-19)

**Authors:** Angela Mazzeo, Enrico Jardim Clemente Santos

**Affiliations:** 1 Brasil Instituto Israelita de Ensino e Pesquisa Albert Einstein, Hospital Israelita Albert Einstein, São Paulo, SP, Brasil.; 2 Brasil Celltrovet-Células Tronco Veterinárias, São Paulo, SP, Brasil.

Caro Editor,

A terapia com células-tronco tem sido alvo de intensas pesquisas básicas, pré-clínicas e clínicas, tendo sido sua segurança e eficácia comprovadas.^(1-4)^ Em estudo realizado em dez pacientes confirmados para doença pelo coronavírus 2019 (COVID-19) acometidos por quadro de pneumonia decorrente da infecção pelo coronavírus da síndrome respiratória aguda grave 2 (SARS-CoV-2), foi observada melhora significativa nos sintomas de todos os pacientes submetidos à aplicação das células-tronco mesenquimais (CTMs) sem a detecção de efeitos adversos. Sete deles foram submetidos à administração endovenosa na concentração de 1x10^6^ células por quilograma de peso corporal e três utilizaram placebo. Os pacientes foram acompanhados por 14 dias, tendo sido observados: significativa redução da infiltração torácica, diminuição da citocina pró-inflamatória fator de necrose tumoral-alfa (TNF-α) eaumento da taxa de linfócitos periféricos com a restauração fenotípica das células T CD4^+^ e dendríticas, além de um aumento da expressão gênica de fatores anti-inflamatórios e tróficos.^(5)^ Embora sejam necessários estudos com maior número de pacientes, os dados já publicados sugerem que as CTMs podem propiciar tratamento seguro e eficaz para os pacientes que apresentam quadro de pneumonia decorrente da infecção pelo SARS-CoV-2.^(5)^

*Chinese Clinical Trial Registry* (ChiCTR2000029990).
